# Association Between the Presence of Missed Canals, Detected Using CBCT, and Post-Treatment Apical Periodontitis in Root-Filled Teeth: A Systematic Review and Meta-Analysis

**DOI:** 10.3390/jcm14165781

**Published:** 2025-08-15

**Authors:** María León-López, Paloma Montero-Miralles, Daniel Cabanillas-Balsera, Juan J. Saúco-Márquez, Jenifer Martín-González, Juan J. Segura-Egea

**Affiliations:** Endodontic Section, Department of Stomatology, School of Dentistry, University of Sevilla, C/Avicena s/n, 41009 Sevilla, Spain; maria.leon.lopez.98@gmail.com (M.L.-L.); dcabanillas@us.es (D.C.-B.); jjsauco@us.es (J.J.S.-M.); jmartin30@us.es (J.M.-G.)

**Keywords:** apical periodontitis, cone beam computed tomography, meta-analysis, missed root canal, root canal treatment failure, root filled teeth, systematic review

## Abstract

**Background.** Post-treatment apical periodontitis (PAP) is a frequent consequence of root canal treatment (RCT) failure, often caused by untreated missed canals in root-filled teeth (RFT). While cone-beam computed tomography (CBCT) has been used to find these missed canals, the results are controversial. This systematic review and meta-analysis investigates the association between PAP in RFT and missed canals detected via CBCT. **Methods**. Two independent reviewers searched PubMed, Scopus, Dialnet, and SciELO for relevant articles published up until 17 February 2025. The main outcome was the prevalence of PAP in RFT with and without missed canals detected via CBCT. The overall odds ratio (OR) was calculated using a binary random effects model meta-analysis (OpenMeta Analyst). Risk of bias was assessed using the Newcastle–Ottawa Scale, and certainty was evaluated using GRADE. **Results**. Eight cross-sectional studies (9983 RFT) were included in the review. The pooled prevalence of PAP was significantly higher in RFT with missed canals (85.1%) than those without (56.3%). The meta-analysis showed a strong association between missed canals and PAP (OR = 7.17, 95% CI = 4.55–11.29), indicating a sevenfold increased likelihood. Maxillary molars, especially first molars, most commonly had missed canals. Heterogeneity was high (I^2^ = 86%), and evidence certainty was low, due to methodological limitations. **Conclusions.** Untreated missed canals significantly increase the likelihood of PAP in RFT, highlighting the need for thorough canal detection and treatment. Clinicians should prioritize anatomical knowledge and advanced imaging to minimize treatment failure.

## 1. Introduction

Apical periodontitis (AP) is an inflammatory condition of the periapical tissues, caused by a bacterial infection within the root canal system. If left untreated, AP persists over time, causing microorganisms to invade the periradicular tissues [1]. The endodontic therapy indicated for managing AP is root canal treatment (RCT) [2]. In these cases, the objective of RCT is to eliminate necrotic dental pulp, seeking to heal periapical tissues [3]. Considering that AP is a very prevalent disease, 52% of people have at least one tooth with AP [4], RCT is a very common endodontic therapy, with the prevalence of RFT being very high worldwide (8%), with 56% of people having at least one RFT [5].

RCT failure leads to post-treatment AP (PAP), which may be due to a persistent, secondary, or extra-radicular infection [6,7]. PAP is radiologically characterized by a periapical radiolucent image, associated with the root apex of an RFT [8].

Several studies have investigated the prevalence of RFT with periapical lesions, and a systematic review with meta-analysis has determined that the prevalence of PAP is high, with 41.3% of RFT showing radiolucent periapical lesions [9,10,11].

The factors causing PAP have been widely investigated [8,12]. One of the main risk factors implicated in RCT failure is inadequate RCT [13,14,15,16,17], with missed canals being one of the most common causes of failed RCT [8,18,19]. Missed untreated canals cause bacteria to persist in the root canal system, leading to the appearance of PAP [20] and causing treatment failure [6,21].

Most of the studies investigating the prevalence of PAP have used periapical radiographs [9,11,22,23] or orthopantomography [24,25,26]. However, these radiograph techniques have a main limitation: they are two-dimensional radiographs [24] and can only detect PAP when there is perforation in the cortical bone [27]. Moreover, when using periapical radiography and orthopantomography it is very difficult to identify untreated canals. With the advent of cone-beam computed tomography (CBCT), three-dimensional radiological information is available, especially when performed at a high resolution, which is very useful for diagnosis, treatment planning, and outcome assessment [28]. Therefore, PAP can be more easily detected using CBCT, with the added advantage that it can not only confirm the presence of the pathology, but it can also identify the cause, such as the presence of missed canals, showing their exact location [20,27]. Beyond its role in detecting untreated canals and periapical pathology, CBCT has also been applied to evaluate the effectiveness of endodontic disinfection protocols, including the identification of vapor lock phenomena that can restrict irrigant penetration and contribute to persistent intraradicular infection, despite the existence of technically adequate instrumentation. This phenomenon, recently documented by Puleio et al. [28], reinforces the broader utility of CBCT in elucidating the multifactorial causes of post-treatment disease.

Several studies have used CBCT to analyze the presence of untreated missed canals in RFT with PAP, which have included controversial results [18,19,29,30,31]. Therefore, this study aimed to perform a systematic review and meta-analysis to investigate the association between the prevalence of PAP and the presence of missed canals, detected using CBCT.

## 2. Materials and Methods

This systematic review was carried out using the PRISMA guidelines and the PECO framework [32,33]. This systematic review has been registered in PROSPERO (CRD420251034983).

### 2.1. Review Question

The research question was as follows: In human adults with RFT (P), the presence of untreated missed canals, detected using CBCT (I), or their absence (C), affects the prevalence of PAP (O)? The question was formulated according to the PECO strategy, as follows:

Population (P): human adults with RFT;

Exposure (E): presence of missed canals diagnosed using CBCT;

Comparison (C): absence of missed canals diagnosed using CBCT;

Outcome (O): prevalence of PAP.

### 2.2. Eligibility Criteria

According to the PECO question, the inclusion/exclusion criteria are outlined below.

#### 2.2.1. Inclusion Criteria

(1) Clinical studies assessing the prevalence of PAP in human RFT and (2) investigating the presence or absence of untreated missed canals using CBCT.

#### 2.2.2. Exclusion Criteria

(1) Clinical studies not reporting the prevalence of PAP in RFT, (2) clinical studies not investigating the presence of untreated missed canals in RFT, (3) clinical studies that did not use CBCT, (4) studies performed on animals, (5) experimental studies that included artificial teeth, and (6) reviews, letters, opinion articles, or conference abstracts.

### 2.3. The Literature Search Strategy and Study Selection

The search process was carried out independently by two reviewers (M.L.-L. and P.M.-M.). Searches were conducted using the electronic databases PubMed, Scopus, Dialnet, and SciELO for articles published up until 17 February 2025, with no restrictions on language, year, or other limits. The most frequently cited descriptors from previous publications on this topic were used to develop the search strategy, combining Medical Subject Headings (MeSH) terms with text words. For each database, the following combinations of terms were used: (prevalence OR frequency) AND (periapical lesion OR apical periodontitis OR radiolucent periapical lesion) AND (missed canals OR untreated canals) AND (root-filled teeth OR endodontically treated teeth) AND (cone-beam computed tomography OR CBCT). Additionally, a supplementary screening of the references from the selected studies was performed to identify any additional studies not found in the primary database search. A search was conducted of the gray literature, but it did not provide any useful data (URL: https://opengrey.eu/; accessed on 10 January 2025; URL: https://scholar.google.com/; accessed on 10 January 2025; and URL: https://www.greynet.org/, accessed on 10 January 2025).

Two independent authors (M.L.-L. and P.M.-M.) selected the retrieved studies by examining the titles and abstracts. When the title and abstract did not allow them to judge the relevance of the study, the full text was accessed. A second stage consisted of reading the full text of the articles and assessing the potential studies to be included based on the eligibility criteria set out as part of the PECO strategy. Disagreements on study inclusion were resolved by consensus with a third author (J.J.S.-E.). Duplicated studies identified during the database search were considered only once.

### 2.4. Data Collection and Extraction Process

Two authors (M.L.-L. and P.M.-M.) collected the data independently from the included studies. A third author (J.J.S.-E.) resolved any disagreements. For each study, the following data were extracted: authors and publication year, study design, sample size, gender and age of the sample, type of teeth studied, distribution of teeth with missed canals, characteristics of CBCT used, and the prevalence of PAP in teeth with and without missed canals.

### 2.5. Data Synthesis and Statistical Analysis

The outcome variable analyzed was the prevalence of PAP in RFT, calculated as the percentage of RFT with radiolucent periapical lesions. In each selected study, the OR was calculated with its 95% confidence interval (CI), to measure the effect of the association between the presence of missed canals and the prevalence of PAP in RFT.

Regarding the overall OR, and its 95% CI, for the prevalence of PAP lesions in RFT, a binary random effects model meta-analysis was carried out, using RevMan software (Review Manager Web. The Cochrane Collaboration, 2019) version 5.4. To estimate the variance and heterogeneity of the studies, the Tau^2^ and Higgins I^2^ tests were used, with slight heterogeneity identified if it ranged from 25% to 50%, moderate heterogeneity if it ranged from 50% to 75%, and high heterogeneity if it was greater than 75% [34]. The level of significance was set to *p* = 0.05.

### 2.6. Quality Assessment and Risk of Bias of Individual Studies

Each selected study was evaluated for its inner methodological risk of bias independently by three authors (J.J.S.-E., M.L.-L., and P.M.-M.). In the case of disagreements over the risk of bias, the article was discussed until a consensus was achieved. The risk of bias in the included studies was evaluated using the Newcastle–Ottawa Scale, adapted for cross-sectional studies [5,35]. This scale was adapted to the outcome of interest, classifying the item into three domains: sample selection, comparability, and outcome. They were given a point (*) depending on whether the aspect required was present or missing. The following criteria were used to evaluate each section:

- Domain “Sample selection” (maximum = 3 points).
  ○ Representativeness of the sample: random sampling → two points; non-random sampling or selected group of patients → one point; no explanation of the sampling selection → no points.
  ○ Sample size: the methods for sample size calculation are provided, or the entire population was enlisted (with a loss rate ≤ 20%) → one point; sample size calculation not provided → no points.
- Domain “Comparability” (maximum = 3 points).
  ○ Control for quality of root canal treatment: Studies that assessed the quality of root canal fillings or coronal restorations and adjusted for it → one point; not controlling for RCT quality → no points.
  ○ Control for other confounding factors (tooth type, age/sex): If the study controlled for a second confounding factor → one point; not controlling for other confounding factors: no points.
  ○ Voxel size of the CBCT: If the analyzed CBCT had a voxel size equal to or smaller than 150 µm → one point; if the analyzed CBCT had a voxel size greater than 150 µm → no points.
- Domain “Outcome” (maximum = 4 points).
  ○ Evaluation of the outcome: If diagnosis criteria for PAP was specified → one point; PAP was diagnosed without specifying criteria → no points.
  ○ Number of observers: two or more → one point; only one → no points.
  ○ CBCT observer experience: If the observer was calibrated and experienced in endodontics → two points; if the observer was not calibrated/or was not experienced in the field of endodontics → one point; if the article did not explain the observer’s calibration or experience → no points.

The lowest possible risk of bias was given a score of 10 points. Studies with scores from 0 to 3 points were considered to be at a high risk of bias, those with scores between 4 and 7 points were considered to be at moderate risk of bias, and, finally, studies with scores between 8 and 10 points were considered to be at low risk of bias.

### 2.7. Grading Recommendations Assessment, Development, and Evaluation

The Grading of Recommendations Assessment, Development, and Evaluation (GRADE) tool was used to assess the overall quality and certainty of the evidence [36]. Four investigators (M.L.-L., P.M.-M., J.J.S.-E., and D.C.-B.) independently carried out the assessment. An initial level of certainty was defined according to the design of the included studies. Then, different domains were analyzed, such as the risk of bias, inconsistency, indirectness, imprecision, publication bias, dose–response gradient, confounding factors, or the magnitude of the effect, finishing with the assignment of a final level of certainty. A high or moderate level of certainty indicates that the true effect probably is close to the estimated conclusion. On the contrary, a low or extremely low level of certainty indicates that confidence in the result is limited or extremely weak, respectively [37].

## 3. Results

### 3.1. Study Selection

**Figure 1:**
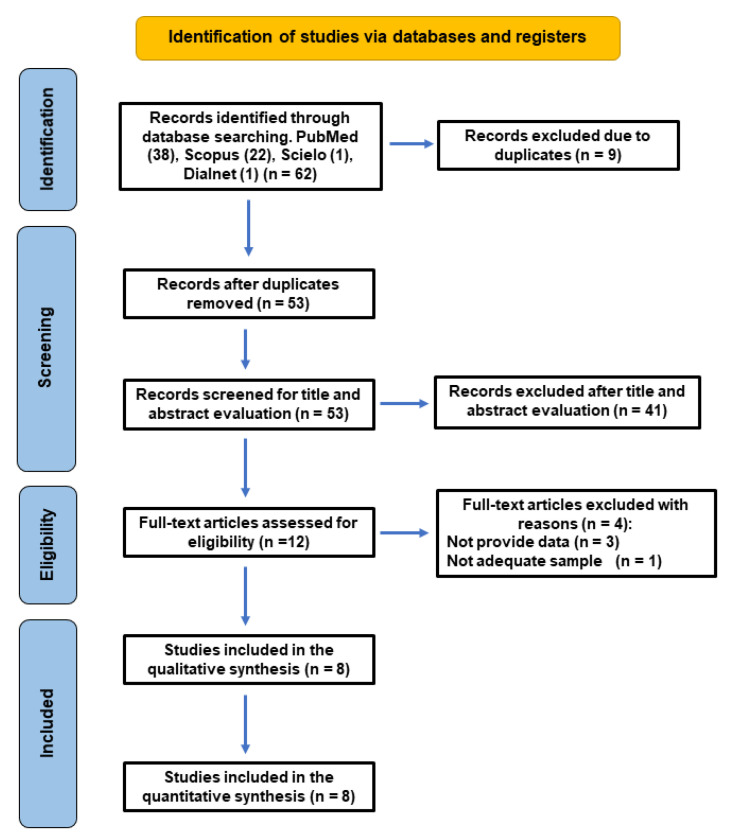
Flowchart of the search strategy, following the PRISMA 2020 guidelines.

Figure 1 shows the flowchart of the search strategy performed. An initial search of the different databases yielded a total of 62 published studies.

After the removal of duplicates, 53 articles remained, of which 41 were excluded after the evaluation of their titles and abstracts for being unrelated to the topic. Twelve articles were assessed for eligibility. After a comprehensive reading of the twelve full-text articles, four were excluded for the following reasons: three did not provide the necessary data on the control group [31,38,39] and another one [40] was excluded because the sample only focused on mesio-buccal canals in maxillary molars.

Then, eight articles [18,19,41,42,43,44,45,46] were selected for the systematic review and meta-analysis.

### 3.2. Characteristics of the Included Studies

The main characteristics of the studies included in the systematic review are shown in Table 1. All of them were cross-sectional studies, examining, using CBCT, the presence of missed canals in RFT, with or without the concurrent presence of PAP.

Regarding the sample size, the study with the smallest sample size was that of Rouhani et al. [44], with 298 patients and 772 RFT, while the one with the largest sample was that of Baruwa et al. [42], with 1160 patients and 2305 RFT. Seven studies provided the patients’ gender distribution [19,41,42,43,44,45,46].

The included studies analyzed different types of RFT: four studies examined all dental groups [19,42,43,46], three studies focused solely on premolars and molars [18,41,45], and one study exclusively analyzed molars [44].

Most studies diagnosed PAP when disruption of the hard lamina and a radiolucency > 2 times the width of the periodontal ligament were present [18,19,41,42,43,44,45]. Only one study used the CBCT PAI index [27] to diagnose PAP [46].

The prevalence of PAP in RFT ranged from 15.8% [41] to 87.6% [19], with most studies found to cite values between 55 and 60% [18,29,43,45].

Concerning the distribution of RFT with missed canals, maxillary first and second molars were the teeth in which the greatest number of missed canals were found, exceeding 50% in most studies [19,42,44,45,46].

Cone-beam computed tomography (CBCT) was used in all the studies, as the radiographic method used to assess the presence of missed canals. The resolution and voxel size varied among the studies: one study used a voxel size of 76 µm [18], two studies used a voxel size of 125 µm [41,46], one study employed a voxel size of 160 µm [44], and four studies utilized a voxel size of 200 µm [19,42,43,45].

### 3.3. Data Extracted from the Included Studies

An evidence table was created to display the data from the included studies related to the prevalence of PAP in RFT both in the presence and absence of untreated missed canals (Table 2).

The prevalence of PAP in RFT with missed canals and in RFT without missed canals was calculated, as well as the corresponding OR for each study and the overall sample. All the studies provided significant OR values, in all cases greater than 3.5, indicating a strong association between the presence of missed canals and the prevalence of PAP. The eight studies included a total of 9983 RFT, presenting 1413 (14.2%) missed canals and 8570 (85.8%) without missed canals. RFT with missed canals showed a PAP prevalence of 85.1%, much higher than that found in RFT without missed canals (56.3%).

### 3.4. Meta-Analysis of the Prevalence of Post-Treatment Apical Periodontitis

**Figure 2:**
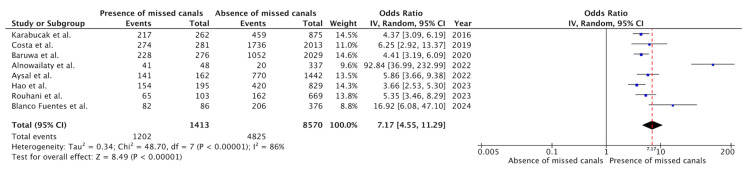
Forest plot of the OR and its 95% confidence interval comparing the prevalence of post-treatment apical periodontitis (PAP) in RFT with and without missed canals. The estimate was based on data from the eight selected studies [18,19,41,42,43,44,45,46].

All the studies included in the systematic review provided the necessary data to carry out the meta-analysis. Thus, eight studies were included in the meta-analysis [18,19,41,42,43,44,45,46], comprising 9983 RFT, to assess whether the presence of a missed canal influenced the prevalence of PAP. The forest plot is shown in Figure 2.

The variance across all of the outcomes was examined using the Tau^2^ test, which revealed significant results (Tau^2^ = 0.34; Chi^2^ = 48.70; df = 7; *p* < 0.00001). The heterogeneity test (I^2^ = 86%) indicated a high level of variability. The overall odds ratio (OR) was 7.17 (95% CI = 4.55–11.29; *p* < 0.00001), indicating a significantly higher prevalence of PAP in teeth with untreated canals, which were seven times more likely to have PAP.

### 3.5. Risk of Bias Assessment

According to the Newcastle–Ottawa Scale, the risk of bias was evaluated for each study (Table 3). One of the included studies was classified as a low risk of bias [46], and the other seven studies were classified as having moderate risk of bias [18,19,41,42,43,44,45].

**Figure 3:**
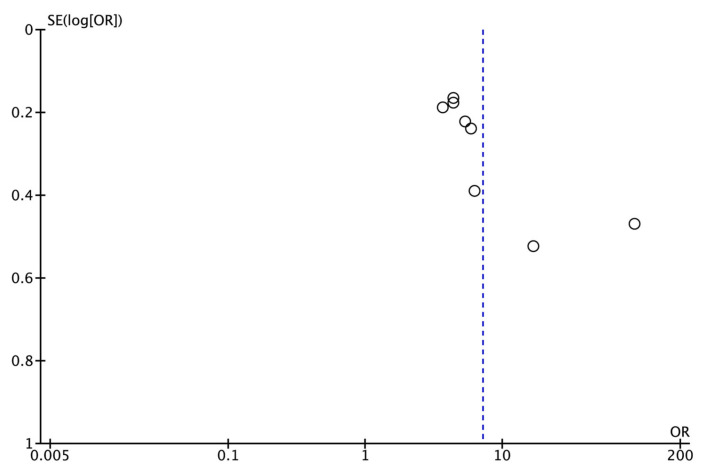
Funnel plot for estimates in the meta-analysis of the prevalence of post-treatment apical periodontitis (PAP) in RFT with and without missed canals. Studies with higher power levels and lower standard errors are placed toward the top. Studies with lower power levels are placed toward the bottom. This blue dotted line indicate the overall effect.

Publication bias could not be assessed quantitatively as there were fewer than the required minimum of 10 studies [34]. However, a funnel plot was plotted to illustrate the possible existence of publication bias (Figure 3).

### 3.6. GRADE Evaluation of Certainty

**Figure 4:**
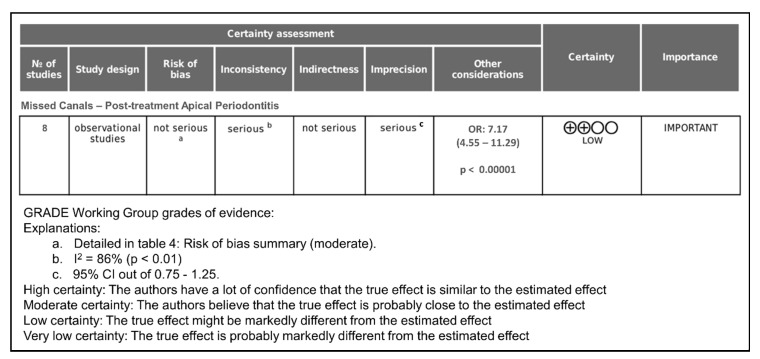
Grade assessment of certainty level (GRADE Working Group).

The certainty of evidence was assessed using the GRADE tool (Figure 4).

All of the articles included were observational studies; therefore, the initial level of certainty was low. The risk of bias domain, according to the overall result (moderate), was classified as “not serious”. The inconsistency domain was considered “serious” as the heterogeneity was high (I^2^ = 86%). All of the studies correctly assessed, reliably the presence of PAP and used CBCT to detect missed canals; without performing indirect comparisons, the indirectness domain was scored as “not serious”. However, the domain of imprecision was rated “serious”, since the 95% CI of the estimated effect (OR) was outside of the 0.75–1.25 range, and the number of included studies was moderate (eight studies).

Publication bias could not be assessed quantitatively due to the low number of studies included, and the funnel plot did not indicate that it was appropriate to downgrade the quality of the evidence. In fact, studies with variable sample sizes that were not funded by the private sector were included in this systematic review.

Finally, it is important to take into account the magnitude of the effect (OR = 7.17; *p* < 0.00001), and that some studies controlled for RCT quality and tooth type. These considerations can raise the degree of certainty by one level. Consequently, the certainty of the evidence was considered low, indicating that the overall OR obtained could differ widely from the real one. The evidence suggests a significant association (OR: 7.17), but the certainty is low due to methodological limitations.

## 4. Discussion

The aim of this systematic review and meta-analysis was to analyze the available evidence about the association between the prevalence of PAP in RFT and the presence of missed canals, detected using CBCT. The findings reveal a strong and statistically significant correlation between the presence of untreated canals and the occurrence of PAP, underscoring the clinical and epidemiological importance of missed canals in regard to endodontic treatment outcomes.

The analysis, based on 9983 RFT across eight cross-sectional studies, found an overall OR of 7.17 (*p* < 0.00001), indicating that teeth with missed canals are over seven times more likely to develop PAP than those in which all the canals have been adequately treated. This OR value implies a high strength of association between both variables and suggests that root canal omission is one of the main factors involved in the development of PAP and in RCT failure. However, the grade of certainty is low due to methodological limitations.

### 4.1. Interpretation of the Main Findings

The central finding of this review confirms what many clinicians have long suspected: missed canals significantly compromise the outcome of endodontic treatment. The high OR, suggesting a strong association [47], and the consistency of this finding across all of the included studies, reinforce the notion that the failure to detect and treat root canals during RCT contributes markedly to the development of PAP. Even though the absolute prevalence of missed canals in RFT was relatively low (14.2%), their presence dramatically increased the risk of pathology.

The prevalence of PAP in teeth with missed canals reached an alarming 85.1%, compared to 56.3% in teeth without untreated canals. While the prevalence of PAP in the latter group is also concerning, suggesting other contributing factors, such as poor obturation, reinfection, or defective coronal restoration, missed canals emerge as a leading and preventable cause of post-treatment failure.

These results are in line with earlier studies [6,8] that highlighted the impact of residual intracanal infection on periapical health.

### 4.2. Diagnostic Superiority of CBCT

One of the strengths of the studies included in this review is the use of CBCT as the diagnostic tool for identifying both PAP and missed canals. Traditional two-dimensional imaging modalities, such as periapical radiographs and orthopantomography, have significant limitations in terms of detecting both periapical pathology and complex root canal anatomies, especially when cortical bone is intact [27,28]. CBCT, by contrast, provides volumetric data, enabling clinicians to visualize the tooth and its surrounding structures in three dimensions and from multiple angles.

The studies analyzed in this meta-analysis employed CBCT units with voxel sizes ranging from 76 µm to 200 µm. Smaller voxel sizes, such as 76 µm [18], offer superior resolution and likely improve the detection of small or complex canal structures. This underscores the role of high-resolution CBCT in both clinical and research settings. Notably, the inclusion of high-quality CBCT imaging in all of the studies strengthens the internal validity of the findings and helps mitigate the risk of diagnostic misclassification.

### 4.3. Anatomical Considerations and Canal Omission

The distribution of missed canals was not random; rather, it followed predictable anatomical patterns. The maxillary first and second molars were the most affected teeth, as reported consistently across the included studies [19,44,46]. This aligns with previous findings in the literature [30,48,49,50], which documents the high frequency of second mesio-buccal canals (MB2) in maxillary molars, canals that are often overlooked due to their narrow diameter, complex morphology, and challenging location.

This pattern emphasizes the importance of thorough knowledge of root canal anatomy and the use of adjunctive technologies, such as CBCT, magnification (e.g., dental loupes or operating microscopes), ultrasonic tips, and dyes to locate hidden or additional canals. As many missed canals occur in anatomically complex teeth, the clinician’s expertise and familiarity with anatomical variations are critical to improving treatment outcomes.

### 4.4. Quality of Endodontic Treatment

While the primary focus of this study was the presence or absence of untreated canals, it is important to acknowledge that other aspects of RCT quality, such as the length and density of obturation, the presence of voids, the coronal seal, and the restoration type, also affect treatment success and the prevalence of PAP.

Although some studies in the review controlled for these variables [42,46], the majority did not. This lack of control for confounding factors limits the ability to isolate missed canals as the sole cause of PAP.

The Newcastle–Ottawa Scale (NOS), adapted for cross-sectional studies [5,35], was used to assess the risk of bias in the included studies. The NOS was adjusted for the purposes of this review by incorporating specific criteria relevant to CBCT-based studies, including the voxel size (≤150 µm vs. >150 µm), calibration and expertise of the CBCT observers, and the specification of PAP diagnostic criteria. These adjustments were intended to reflect methodological aspects that could influence the detection of missed canals and the diagnosis of PAP in CBCT imaging.

The NOS revealed that only one study had a low risk of bias, while the remaining seven were classified as moderate risk. This suggests that while the overall evidence supports a strong association between missed canals and PAP, methodological limitations may influence the strength of that conclusion. Future studies should aim to control known confounding variables and include more rigorous criteria for assessing root filling and coronal restoration quality.

### 4.5. Methodological Strengths and Limitations

One of the notable strengths of this meta-analysis lies in its strict inclusion criteria and comprehensive search strategy, which included multiple databases and supplementary manual searches. All of the included studies met predefined eligibility criteria, used CBCT as the imaging modality, and provided sufficient data to calculate ORs for PAP in the presence and absence of missed canals.

Nonetheless, some limitations must be acknowledged. First, all of the included studies were cross-sectional, meaning that temporal causality cannot be definitively established. While the strong association between missed canals and PAP suggests a causal relationship, longitudinal cohort studies are needed to confirm that missed canals precede and lead to PAP rather than being incidental findings.

Second, heterogeneity among the studies was substantial (I^2^ = 86%). This was likely due to differences in the sample size, voxel size of CBCT, definitions of PAP, geographic populations, and the inclusion of different types of teeth. Although a random effects model was used to accommodate variability, the wide confidence intervals in some studies [41] indicate uncertainty that may impact the pooled estimate.

Third, the overall level of certainty, as assessed using the GRADE tool, was considered low. This was primarily due to imprecision and inconsistency, rather than serious risk of bias or indirectness. The magnitude of the effect, however (OR > 7), is substantial enough to suggest that the association is unlikely to be due to chance alone [47].

Finally, the presence of potential publication bias could not be excluded. With fewer than 10 studies, formal tests for funnel plot asymmetry lack power and were not applicable. However, diverse study inclusion from various regions and sample sizes likely reduces the selective reporting risks.

Another methodological consideration is the variability in the CBCT voxel size among the included studies, ranging from 76 µm to 200 µm. Smaller voxel sizes may enhance the detection of narrow or complex canal systems, as well as subtle periapical changes, whereas larger voxel sizes might underestimate their presence. Moreover, the diagnostic criteria for PAP were not standardized across the studies; most relied on radiolucency size thresholds or hard lamina disruption, while only one employed the CBCT PAI index. This variability may have influenced the prevalence estimates and contributed to the high heterogeneity observed.

Beyond anatomical detection, it is important to acknowledge that root canal treatment outcomes are also shaped by intracanal disinfection efficacy. Incomplete irrigant penetration, often due to vapor lock phenomena, can enable microbial persistence, despite the use of technically adequate instrumentation. CBCT-based imaging has recently been applied to visualize these vapor lock effects [28], providing valuable insight into how such irrigation limitations can contribute to post-treatment apical periodontitis. These findings reinforce the multifactorial nature of endodontic treatment failure and the broader utility of CBCT in regard to its assessment.

### 4.6. Comparison with Previous Literature

RCT failure and the development of PAP is a widely studied topic in the endodontic literature [51,52,53]. The relationship between PAP and the presence of omitted canals has not only been analyzed in the studies included in this systematic review, but also in many other studies [31,38,39]. The findings of this meta-analysis are congruent with prior studies that identified missed canals as a common cause of endodontic failure [8,54]. Siqueira et al. [55]. reported that missed canal systems are among the most frequent causes of retreatment. Chugal et al. [56] emphasized the role of an untreated intracanal infection in the persistence of periapical pathology.

A systematic review [57] found that approximately 41.3% of RFT had periapical lesions, regardless of whether canals were missed or not, which is consistent with the baseline PAP prevalence found in teeth without missed canals in the current study. Thus, while missed canals are a significant contributor, other factors clearly play a role in the etiology of PAP.

Moreover, it has been recently demonstrated that untreated canals are a significant predictor of endodontic failure over a 5-year period, further supporting the findings of this meta-analysis [58]. These studies collectively reinforce the clinical importance of complete debridement and obturation of all of the canals present to ensure long-term success.

### 4.7. Clinical Implications

The clinical implications of this review are substantial. Endodontic success depends heavily on the ability to thoroughly clean, shape, and obturate the entire root canal system. Missing a canal not only allows microbial biofilms to persist, but also creates an inaccessible reservoir for reinfection, potentially leading to chronic inflammation, symptom recurrence, and treatment failure.

For general dentists and endodontists alike, this review emphasizes the need for meticulous diagnostic evaluation prior to and during RCT. When anatomical complexities are suspected, especially in maxillary molars, CBCT should be considered a valuable adjunct. Moreover, the routine use of magnification and illumination, as well as updated instrumentation protocols, should be encouraged to minimize the risk of missed canals.

The findings also underscore the value of post-treatment assessment. CBCT imaging can be useful not only for diagnosis, but also for evaluating the success of the treatment, particularly when symptoms persist or when planning retreatment. Given the high risk of PAP associated with untreated canals, clinicians must maintain a high index of suspicion and consider early radiographic re-evaluation when outcomes are uncertain.

### 4.8. Public Health and Educational Impact

From a public health perspective, the high prevalence of PAP in RFT, whether due to missed canals or other factors, represents a burden both for healthcare systems and for patients. PAP often progresses silently and may go unnoticed until the onset of acute symptoms, at which point treatment becomes more complex and costly. The need for retreatment, periapical surgery, or extraction can be avoided through higher initial treatment quality.

These findings also have implications for dental education. Curricula should emphasize the anatomical variations and techniques for locating hidden canals, as well as the integration of advanced diagnostic tools. Dental schools must equip students with both the theoretical knowledge and clinical skills necessary to prevent canal omission.

In addition, continuing education programs for practicing clinicians should reinforce the importance of recognizing high-risk cases and utilizing CBCT judiciously to improve diagnostic accuracy.

### 4.9. Recommendations for Future Research

Future research should aim to address some of the limitations of the present evidence. Longitudinal cohort studies that follow patients over time after RCT and assess the incidence of PAP in relation to missed canals would offer stronger causal inferences. Additionally, randomized controlled trials (where ethically feasible) could explore the benefits of adjunctive CBCT use during diagnosis or retreatment planning.

Further studies should also aim to standardize the criteria for diagnosing PAP using CBCT, perhaps by universally adopting validated indices, such as the CBCT PAI index [27]. Additionally, investigators should control for confounding variables, such as obturation quality, coronal seal, patient age, systemic conditions, and tooth type.

Finally, economic evaluations would be valuable to assess the cost effectiveness of CBCT imaging in reducing long-term failure rates and avoiding costly retreatments. Such evidence could guide health policy and reimbursement decisions for diagnostic imaging in endodontics.

## 5. Conclusions

Post-treatment apical periodontitis is associated with the presence of a missed root canals in RFT. The presence of untreated missed canals significantly increases the likelihood of PAP in RFT, underscoring the importance of thorough canal detection and treatment. Clinicians should prioritize increasing their anatomical knowledge and the use of advanced imaging to reduce treatment failure.

## Figures and Tables

**Table 1:** Characteristics of the included studies.

Authors and Year	Country	Study Type	Sample (%)	Types of Included RFT	Prevalence of PAP in RFT (%)	Presence of Missed Canals (%)	CBCT Voxel Size
Karabucak et al., 2016 [18]	USA	Cross-sectional	655 patients 1137 RFT	Molar and premolar	59.5 *	Maxillary molars (40.1) Maxillary premolars (9.5)	76 µm
Costa et al., 2019 [19]	Brazil	Cross-sectional	764 patients 2294 RFT Men (37) Women (63)	All	87.6 *	Maxillary molars (57) Mandibular molars (26) Maxillary premolars (10) Mandibular premolars (6)	200 µm
Baruwa et al., 2020 [42]	Portugal	Cross-sectional	1160 patients 2305 RFT Men (43) Women (57)	All	55.5 *	Maxillary 1st molars (59.5) Maxillary 2nd molars (40) Mandibular 1st molars (11.2) Mandibular 2nd molars (9.5)	≤200 µm
Alnowailaty & Alghamdi et al., 2022 [41]	Saudi Arabia	Cross-sectional	300 patients 385 RFT Men (50) Women (50)	Molar and premolar	15.8 *	Maxillary teeth (26) Mandibular teeth (22) Maxillary 2nd molars (38.1)	125 µm
Aysal et al., 2022 [43]	Turkey	Cross-sectional	1069 patients 1604 RFT Men (51) Women (49)	All	56.8 *	Teeth without apical lesions (3) Teeth with apical lesions (15.5)	≤200 µm
Hao et al., 2023 [45]	China	Cross-sectional	561 patients 1024 RFT Men (35) Women (65)	Molar and premolar	56.1 *	Maxillary 1st molars (65.7) Mandibular 1st molars (16.4) Mandibular 2nd molars (11.7) Maxillary 2nd molars (10.3)	200 µm
Rouhani et al., 2023 [44]	Iran	Cross-sectional	298 patients 772 RFT Men (46) Women (54)	Molar	29.4 *	Maxillary 1st molars (56) Maxillary 2nd molars (14) Mandibular 1st molars (10)	160 µm
Blanco Fuentes et al., 2024 [46]	Colombia	Cross-sectional	318 patients 462 RFT Men (40) Women (60)	All	62.3 **	Maxillary 1st molars (61.9) Maxillary 2nd molars (41.1) Mandibular 1st molars (18.8)	125 or 200 µm

PAP: post-treatment apical periodontitis; RFT: root-filled teeth; * PAP diagnosed as disruption of the hard lamina and a radiolucency > 2 times the width of the periodontal ligament; ** PAP diagnosed using CBCT PAI index.

**Table 2:** Prevalence of PAP in RFT in relation to the presence or absence of missed canals.

Authors and Year	RFT (No.)	Presence of Missed Canals		Absence of Missed Canals		Odds Ratio (95% CI)	*p*
		RFT	Prevalence of PAP (%)	RFT	Prevalence of PAP (%)		
Karabucak et al., 2016 [18]	1137	262	217 (82.8)	875	459 (52.5)	4.37 (3.1–6.2)	<0.01
Costa et al., 2019 [19]	2294	281	274 (97.5)	2013	1736 (86.2)	6.25 (2.9–13.4)	<0.01
Baruwa et al., 2020 [42]	2305	276	228 (82.6)	2029	1052 (51.8)	4.41 (3.2–6.1)	<0.01
Alnowailaty & Alghamdi et al., 2022 [41]	385	48	41 (85.4)	337	20 (5.9)	92.84 (37.0–233.0)	<0.01
Aysal et al., 2022 [43]	1604	162	141 (87.0)	1442	770 (53.4)	5.86 (3.7–9.4)	<0.01
Hao et al., 2023 [45]	1024	195	154 (79.0)	829	420 (50.7)	3.66 (2.5–5.3)	<0.01
Rouhani et al., 2023 [44]	772	103	65 (63.1)	669	162 (24.2)	5.35 (3.5–8.3)	<0.01
Blanco Fuentes et al., 2024 [46]	462	86	82 (95.3)	376	206 (54.8)	16.92 (6.1–47.1)	<0.01
Overall	9983	1413	1202 (85.1)	8570	4825 (56.3)		

**Table 3:** Assessment of risk of bias according to Newcastle–Ottawa Scale, adapted for cross-sectional studies.

Author and Year	Sample Selection		Comparability			Outcome			Risk of Bias
	Sample Representativeness	Sample Size	Control for RCT Quality	Control for Other Confounding Factors	Voxel Size	Criteria for PAP Diagnosis	No. Observers	CBCT Observer Experience	
Karabucak et al., 2016 [18]	*	-	-	-	*	*	*	*	5 Moderate
Costa et al., 2019 [19]	*	-	-	-	-	*	*	**	5 Moderate
Baruwa et al., 2020 [42]	*	-	*	*	-	*	*	**	7 Moderate
Alnowailaty & Alghamdi et al., 2022 [41]	*	-	-	-	*	*	*	**	6 Moderate
Aysal et al., 2022 [43]	*	-	-	-	-	*	*	*	4 Moderate
Hao et al., 2023 [45]	*	-	-	-	-	*	*	*	4 Moderate
Rouhani et al., 2023 [44]	*	-	-	-	-	*	*	*	4 Moderate
Blanco Fuentes et al., 2024 [46]	*	-	*	*	*	*	*	**	8 Low
OVERALL	8	0	2	2	3	8	8	12	43/80 Moderate

Each * represent one point, depending on whether the aspect required was present or missing, according to Mat & Met.

## Data Availability

No new data were created or analyzed in this study. Data sharing is not applicable to this article.

## References

[1] Ricucci D., Siqueira J.F. (2010). Biofilms and apical periodontitis: Study of prevalence and association with clinical and histopathologic findings. J. Endod..

[2] Duncan H.F., Kirkevang L.L., Peters O.A., El-Karim I., Krastl G., Del Fabbro M., Chong B.S., Galler K.M., Segura-Egea J.J., Kebschull M. (2023). Treatment of pulpal and apical disease: The European Society of Endodontology (ESE) S3-level clinical practice guideline. Int. Endod. J..

[3] Ricucci D., Lin L.M., Spångberg L.S.W. (2009). Wound healing of apical tissues after root canal therapy: A long-term clinical, radiographic, and histopathologic observation study. Oral Surg. Oral Med. Oral Pathol. Oral Radiol. Endodontology.

[4] Tibúrcio-Machado C.S., Michelon C., Zanatta F.B., Gomes M.S., Marin J.A., Bier C.A. (2021). The global prevalence of apical periodontitis: A systematic review and meta-analysis. Int. Endod. J..

[5] León-López M., Cabanillas-Balsera D., Martín-González J., Montero-Miralles P., Saúco-Márquez J.J., Segura-Egea J.J. (2022). Prevalence of root canal treatment worldwide: A systematic review and meta-analysis. Int. Endod. J..

[6] Ricucci D., Siqueira J.F., Bate A.L., Pitt Ford T.R. (2009). Histologic Investigation of Root Canal–treated Teeth with Apical Periodontitis: A Retrospective Study from Twenty-four Patients. J. Endod..

[7] Provenzano J.C., Antunes H.S., Alves F.R.F., Rôças I.N., Alves W.S., Silva M.R.S., Siqueira J.F. (2016). Host-Bacterial Interactions in Post-treatment Apical Periodontitis: A Metaproteome Analysis. J. Endod..

[8] Nair P.N.R. (2006). On the causes of persistent apical periodontitis: A review. Int. Endod. J..

[9] Jiménez-Pinzón A., Segura-Egea J.J., Poyato-Ferrera M., Velasco-Ortega E., Ríos-Santos J.V. (2004). Prevalence of apical periodontitis and frequency of root-filled teeth in an adult Spanish population. Int. Endod. J..

[10] Segura-Egea J.J., Martín-González J., Cabanillas-Balsera D., Fouad A.F., Velasco-Ortega E., López-López J. (2016). Association between diabetes and the prevalence of radiolucent periapical lesions in root-filled teeth: Systematic review and meta-analysis. Clin. Oral Investig..

[11] Sunay H., Tanalp J., Dikbas I., Bayirli G. (2007). Cross-sectional evaluation of the periapical status and quality of root canal treatment in a selected population of urban Turkish adults. Int. Endod. J..

[12] Ricucci D., Russo J., Rutberg M., Burleson J.A., Spngberg L.S.W. (2011). A prospective cohort study of endodontic treatments of 1,369 root canals: Results after 5 years. Oral Surg. Oral Med. Oral Pathol. Oral Radiol. Endodontology.

[13] Siqueira J.F., Rôças I.N. (2005). Exploiting molecular methods to explore endodontic infections: Part 1—current molecular technologies for microbiological diagnosis. J. Endod..

[14] Tavares P.B.L., Bonte E., Boukpessi T., Siqueira J.F., Lasfargues J.J. (2009). Prevalence of Apical Periodontitis in Root Canal-Treated Teeth from an Urban French Population: Influence of the Quality of Root Canal Fillings and Coronal Restorations. J. Endod..

[15] Segura-Egea J.J., Cabanillas-Balsera D., Martín-González J., Cintra L.T.A. (2023). Impact of systemic health on treatment outcomes in endodontics. Int. Endod. J..

[16] Morsani J.M., Aminoshariae A., Han Y.W., Montagnese T.A., Mickel A. (2011). Genetic predisposition to persistent apical periodontitis. J. Endod..

[17] Eriksen H.M., Bjertness E. (1991). Prevalence of apical periodontitis and results of endodontic treatment in middle-aged adults in Norway. Endod. Dent. Traumatol..

[18] Karabucak B., Bunes A., Chehoud C., Kohli M.R., Setzer F. (2016). Prevalence of Apical Periodontitis in Endodontically Treated Premolars and Molars with Untreated Canal: A Cone-beam Computed Tomography Study. J. Endod..

[19] Costa F.F.N.P., Pacheco-Yanes J., Siqueira J.F., Oliveira A.C.S., Gazzaneo I., Amorim C.A., Santos P.H.B., Alves F.R.F. (2019). Association between missed canals and apical periodontitis. Int. Endod. J..

[20] Huumonen S., Suominen A.L., Vehkalahti M.M. (2017). Prevalence of apical periodontitis in root filled teeth: Findings from a nationwide survey in Finland. Int. Endod. J..

[21] Nair P.N.R. (2004). Pathogenesis of Apical Periodontitis and the Causes of Endodontic Failures. Crit. Rev. Oral Biol. Med..

[22] Dugas N.N., Lawrence H.P., Teplitsky P.E., Pharoah M.J., Friedman S. (2003). Periapical health and treatment quality assessment of root-filled teeth in two Canadian populations. Int. Endod. J..

[23] Lõpez-Lõpez J., Jané-Salas E., Estrugo-Devesa A., Castellanos-Cosano L., Martín-González J., Velasco-Ortega E., Segura-Egea J.J. (2012). Frequency and distribution of root-filled teeth and apical periodontitis in an adult population of Barcelona, Spain. Int. Dent. J..

[24] Ríos-Santos J.V., Ridao-Sacie C., Bullón P., Fernández-Palacín A., Segura-Egea J.J. (2010). Assessment of periapical status: A comparative study using film-based periapical radiographs and digital panoramic images. Med. Oral Patol. Oral Cir. Bucal.

[25] Lupi-Pegurier L., Bertrand M.F., Muller-Bolla M., Rocca J.P., Bolla M. (2002). Periapical status, prevalence and quality of endodontic treatment in an adult French population. Int. Endod. J..

[26] Huumonen S., Vehkalahti M.M., Nordblad A. (2012). Radiographic assessments on prevalence and technical quality of endodontically-treated teeth in the Finnish population, aged 30 years and older. Acta Odontol. Scand..

[27] Estrela C., Bueno M.R., Azevedo B.C., Azevedo J.R., Pécora J.D. (2008). A new periapical index based on cone beam computed tomography. J. Endod..

[28] Puleio F., Lizio A.S., Coppini V., Lo Giudice R., Lo Giudice G. (2023). CBCT-Based assessment of vapor lock effects on endodontic disinfection. Appl. Sci..

[29] Pereira B., Martins J.N.R., Baruwa A.O., Meirinhos J., Gouveia J., Quaresma S.A., Monroe A., Ginjeira A. (2020). Association between Endodontically Treated Maxillary and Mandibular Molars with Fused Roots and Periapical Lesions: A Cone-beam Computed Tomography Cross-sectional Study. J. Endod..

[30] Martins J.N.R., Alkhawas M.B.A.M., Altaki Z., Bellardini G., Berti L., Boveda C., Chaniotis A., Flynn D., Gonzalez J.A., Kottoor J. (2018). Worldwide Analyses of Maxillary First Molar Second Mesiobuccal Prevalence: A Multicenter Cone-beam Computed Tomographic Study. J. Endod..

[31] Mashyakhy M., Hadi F.A., Alhazmi H.A., Alfaifi R.A., Alabsi F.S., Bajawi H., Alkahtany M., AbuMelha A. (2021). Prevalence of Missed Canals and Their Association with Apical Periodontitis in Posterior Endodontically Treated Teeth: A CBCT Study. Int. J. Dent..

[32] Moher D., Shamseer L., Clarke M., Ghersi D., Liberati A., Petticrew M., Shekelle P., Stewart L.A., Estarli M., Barrera E.S.A. (2016). Preferred reporting items for systematic review and meta-analysis protocols (PRISMA-P) 2015 statement. Rev. Esp. Nutr. Humana Y Diet..

[33] Page M.J., McKenzie J.E., Bossuyt P.M., Boutron I., Hoffmann T.C., Mulrow C.D., Shamseer L., Tetzlaff J.M., Akl E.A., Brennan S.E. (2021). The PRISMA 2020 statement: An updated guideline for reporting systematic reviews. Syst. Rev..

[34] Higgins J.P.T., Thompson S.G. (2002). Quantifying heterogeneity in a meta-analysis. Stat. Med..

[35] Herzog R., Álvarez-Pasquin M.J., Díaz C., Del Barrio J.L., Estrada J.M., Gil Á. (2013). Are healthcare workers intentions to vaccinate related to their knowledge, beliefs and attitudes? A systematic review. BMC Public Health.

[36] Guyatt G.H., Oxman A.D., Vist G., Kunz R., Brozek J., Alonso-Coello P., Montori V., Akl E.A., Djulbegovic B., Falck-Ytter Y. (2011). GRADE guidelines: 4. Rating the quality of evidence—study limitations (risk of bias). J. Clin. Epidemiol..

[37] Hultcrantz M., Rind D., Akl E.A., Treweek S., Mustafa R.A., Iorio A., Alper B.S., Meerpohl J.J., Murad M.H., Ansari M.T. (2017). The GRADE Working Group clarifies the construct of certainty of evidence. J. Clin. Epidemiol..

[38] Meirinhos J., Martins J.N.R., Pereira B., Baruwa A.O., Ginjeira A. (2021). Prevalence of Lateral Radiolucency, Apical Root Resorption and Periapical Lesions in Portuguese Patients: A CBCT Cross-Sectional Study with a Worldwide Overview. Eur. Endod. J..

[39] do Carmo W.D., Verner F.S., Aguiar L.M., Visconti M.A., Ferreira M.D., Lacerda M.F.L.S., Junqueira R.B. (2021). Missed canals in endodontically treated maxillary molars of a Brazilian subpopulation: Prevalence and association with periapical lesion using cone-beam computed tomography. Clin. Oral Investig..

[40] Alotaibi B.B., Khan K.I., Javed M.Q., Dutta S.D., Shaikh S.S., Almutairi N.M. (2023). Relationship between apical periodontitis and missed canals in mesio-buccal roots of maxillary molars: CBCT study. J. Taibah Univ. Med. Sci..

[41] Alnowailaty Y., Alghamdi F. (2022). Prevalence of Endodontically Treated Premolars and Molars with Untreated Canals and Their Association with Apical Periodontitis Using Cone-Beam Computed Tomography. Cureus.

[42] Baruwa A.O., Martins J.N.R., Meirinhos J., Pereira B., Gouveia J., Quaresma S.A., Monroe A., Ginjeira A. (2020). The Influence of Missed Canals on the Prevalence of Periapical Lesions in Endodontically Treated Teeth: A Cross-sectional Study. J. Endod..

[43] Aysal Z., Kocasarac H.D., Orhan K., Helvacioglu-Yigi D. (2022). Radiological Assessment of Prevalance and Quality of Periapical Status of Endodontic Treatments. Med. Sci. Monit..

[44] Rouhani A., Aboutorabzadeh S.M.R., Reyhani M., Kheirabadi N., Mortazavi S., Navabi S. (2023). Prevalence of missed canals in endodontically treated teeth: A cone-beam computed tomography study. J. Clin. Exp. Dent..

[45] Hao J., Liu H., Shen Y. (2023). Periapical Lesions and Missed Canals in Endodontically Treated Teeth: A Cone-Beam Computed Tomographic Study of a Chinese Subpopulation. Med. Sci. Monit..

[46] Blanco Fuentes B.Y., Moreno Monsalve J.O., Mesa Herrera U., Amoroso-Silva P.A., Rodrigues Ferreira Alves F., Marceliano-Alves M.F. (2024). Apical periodontitis in endodontically-treated teeth: Association between missed canals and quality of endodontic treatment in a Colombian subpopulation. A cross-sectional study. Acta Odontol. Latinoam..

[47] Hill A.B. (1965). The environment and disease: Association or causation?. Proc. R. Soc. Med..

[48] Wolcott J., Ishley D., Kennedy W., Johnson S., Minnich S., Meyers J. (2005). A 5 yr clinical investigation of second mesiobuccal canals in endodontically treated and retreated maxillary molars. J. Endod..

[49] Peña-Bengoa F., Cáceres C., Niklander S.E., Meléndez P. (2023). Association between second mesiobuccal missed canals and apical periodontitis in maxillary molars of a Chilean subpopulation. J. Clin. Exp. Dent..

[50] Studebaker B., Hollender L., Mancl L., Johnson J.D., Paranjpe A. (2018). The Incidence of Second Mesiobuccal Canals Located in Maxillary Molars with the Aid of Cone-beam Computed Tomography. J. Endod..

[51] Ng Y.-L.L., Mann V., Gulabivala K. (2011). A prospective study of the factors affecting outcomes of non-surgical root canal treatment: Part 2: Tooth survival. Int. Endod. J..

[52] Burns L.E., Kim J., Wu Y., Alzwaideh R., McGowan R., Sigurdsson A. (2022). Outcomes of primary root canal therapy: An updated systematic review of longitudinal clinical studies published between 2003 and 2020. Int. Endod. J..

[53] Siqueira J.F., Rôças I.N., Ricucci D., Hülsmann M. (2014). Causes and management of post-treatment apical periodontitis. Br. Dent. J..

[54] Witherspoon D., Small J.C., Regan J.D. Missed Canal Systems Are the Most Likely Basis for Endodontic Retreatment of Molars—PubMed. https://pubmed.ncbi.nlm.nih.gov/23930451/.

[55] Siqueira J.F., Rôças I.N., Alves F.R.F., Campos L.C. (2005). Periradicular status related to the quality of coronal restorations and root canal fillings in a Brazilian population. Oral Surg. Oral Med. Oral Pathol. Oral Radiol. Endod..

[56] Chugal N.M., Clive J.M., Spångberg L.S.W. (2003). Endodontic infection: Some biologic and treatment factors associated with outcome. Oral Surg. Oral Med. Oral Pathol. Oral Radiol. Endod..

[57] Jakovljevic A., Nikolic N., Jacimovic J., Pavlovic O., Milicic B., Beljic-Ivanovic K., Miletic M., Andric M., Milasin J. (2020). Prevalence of Apical Periodontitis and Conventional Nonsurgical Root Canal Treatment in General Adult Population: An Updated Systematic Review and Meta-analysis of Cross-sectional Studies Published between 2012 and 2020. J. Endod..

[58] Jang Y.E., Kim Y., Kim S.Y., Kim B.S. (2024). Predicting early endodontic treatment failure following primary root canal treatment. BMC Oral Health.

